# NO Synthesis Markers Are Not Significantly Associated with Blood Pressure and Endothelial Dysfunction in Patients with Arterial Hypertension: A Cross-Sectional Study

**DOI:** 10.3390/jcm9123895

**Published:** 2020-11-30

**Authors:** Oliver Malle, Christian Trummer, Verena Theiler-Schwetz, Andreas Meinitzer, Martin H. Keppel, Martin R. Grübler, Andreas Tomaschitz, Jakob Voelkl, Winfried März, Stefan Pilz

**Affiliations:** 1 Department of Internal Medicine, Division of Endocrinology and Diabetology, Medical University of Graz, 8036 Graz, Austria; stefan.pilz@chello.at; 2 Clinical Institute of Medical and Chemical Laboratory Diagnostics, Medical University of Graz, 8036 Graz, Austria; christian.trummer@medunigraz.at; 3 Department of Laboratory Medicine, Paracelsus Medical University Salzburg, 5020 Salzburg, Austria; verena.schwetz@medunigraz.at; 4 Department of Internal Medicine, Division of Endocrinology and Diabetology, Endocrinology Lab Platform, Medical University of Graz, 8036 Graz, Austria; andreas.meinitzer@medunigraz.at; 5 Health Center Trofaiach-Gössgrabenstrasse, 8793 Trofaiach, Austria; keppel.martin@gmail.com; 6 Institute for Physiology and Pathophysiology, Johannes Kepler University, 4040 Linz, Austria; martin.gruebler@gmx.net; 7 DZHK (German Centre for Cardiovascular Research), Partner Site Berlin, 10785 Berlin, Germany; andreas.tomaschitz@gmx.at; 8 Department of Nephrology and Medical Intensive Care, Charité—Universitätsmedizin Berlin, 10117 Berlin, Germany; jakob.voelkl@charite.de; 9 Medical Clinic V (Nephrology, Hypertensiology, Rheumatology, Endocrinology, Diabetology), Medical Faculty Mannheim, University of Heidelberg, 68167 Mannheim, Germany; winfried.maerz@synlab.com

**Keywords:** endothelial dysfunction, homoarginine, ADMA, SDMA, NO, arterial hypertension, blood pressure, pulse wave velocity

## Abstract

Nitric oxide (NO) synthesis markers, comprising L-homoarginine, asymmetric dimethylarginine (ADMA) and symmetric dimethylarginine (SDMA), are significantly associated with cardiovascular events and mortality. Being involved in NO pathways, they may be of high importance regulating vascular tone and arterial hypertension, but data on this topic are sparse and controversial. In this study, we evaluated whether these NO synthesis markers are associated with blood pressure values and pulse wave velocity (PWV). This analysis was based on the data of the Styrian Vitamin D Hypertension Trial, which included adults with arterial hypertension. We analyzed correlations of NO synthesis markers with 24 h ambulatory blood pressure values and PWV (primary outcomes), as well as with anthropometric and laboratory data. A total of 509 patients were included in the present analysis. The mean age was 61.2 ± 10.5 years, mean PWV was 8.6 ± 2.4 m/s, mean 24 h systolic blood pressure was 127.5 ± 13.8 mmHg and mean 24 h diastolic blood pressure was 76.4 ± 9.5 mmHg. In bivariate analyses, there was a significant positive correlation between homoarginine and 24 h diastolic blood pressure (r = 0.1; *p =* 0.02), which was revealed to be no longer significant after adjustment for age, gender and glomerular filtration rate (GFR) in multivariate regression analysis. No other significant correlations of any NO synthesis markers with blood pressure or PWV were observed. In line with previous studies, there were inverse associations between homoarginine and age and between ADMA or SDMA and GFR (*p* < 0.05 for all). This study did not reveal a significant association between homoarginine, ADMA or SDMA and blood pressure or PWV in hypertensive adults. These results suggested that the associations of these parameters with adverse outcome may not be mediated by hypertension and/or endothelial dysfunction.

## 1. Introduction

Well-established risk factors in cardiovascular disease (CVD) comprise, inter alia, arterial hypertension, hypercholesterinemia, diabetes mellitus and smoking. CVD management focusing on these modifiable risk factors resulted in a reduction of cardiovascular mortality over the last decades, but it remains the leading cause of death worldwide [1,2]. Nitric oxide (NO) is a potent endogenous vasodilator generated by the enzyme endothelial NO synthase from the substrate L-arginine [3,4]. Low NO levels may contribute to an impairment of vascular system function, resulting in dysregulation of blood pressure as well as endothelial dysfunction [5,6].

Several biochemical parameters with relevance for NO metabolism, i.e., L-homoarginine, asymmetric dimethylarginine (ADMA) and symmetric dimethylarginine (SDMA), may therefore be involved in the pathogenesis of arterial hypertension and endothelial dysfunction. Homoarginine, a homologue of L-arginine, is synthesized enzymatically from L-arginine by arginine:glycine amidinotransferase and possibly also by enzymes of the urea cycle but can also be derived from food intake to an unknown extent [7]. Serving as a substrate for NO synthase and by inhibiting arginase and arginine methyltransferase enzymes, homoarginine may enhance NO synthesis and, hence, may improve endothelial function and exert antiatherosclerotic effects [8]. Homoarginine was shown to be associated with cardiovascular mortality [8,9,10,11,12], congestive heart failure [13], diastolic heart failure [13] and peripheral artery disease [14].

**Figure 1:**
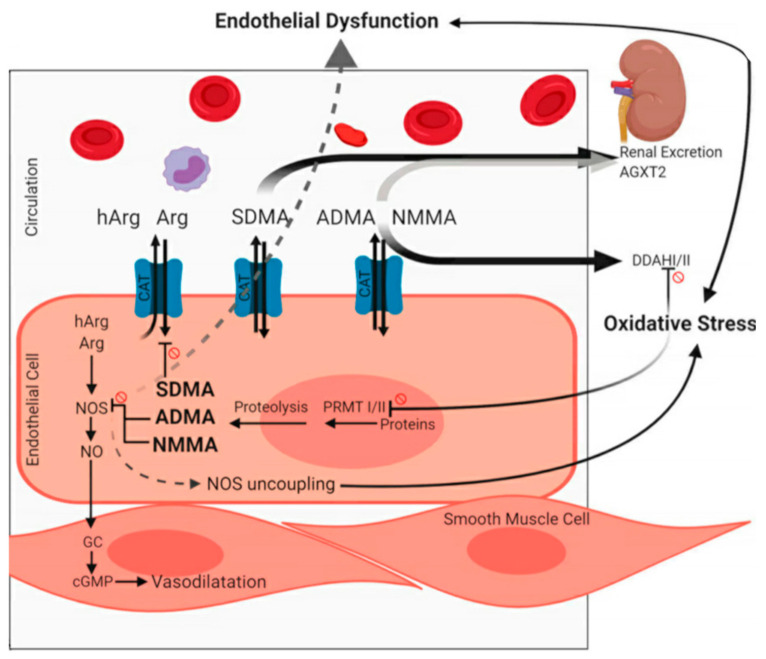
Overview of NO metabolism reproduced from Grosse et al. [28] under the terms of the Creative Common Attribution 4.0 International license. NO: nitric oxide; Arg: arginine (Arg); hArg: homoarginine; ADMA: asymmetric dimethylarginine; SDMA: symmetric dimethylarginine; NMMA: monomethylarginine; AGXT2: Alanine-glyoxylate aminotransferase 2; DDAHI/II: dimethylarginine dimethylaminohydrolase I/II; PRMT I/II: protein arginine methyltransferases I/II; cGMP: Cyclic guanosinmonophosphate. Prohibition signs besides lines refer to an inhibitory relationship.

Asymmetric dimethylarginine (ADMA) and symmetric dimethylarginine (SDMA) are endogenous products and are increasingly being recognized to be involved in the pathophysiology of endothelial dysfunction [15,16,17,18]. ADMA derives from arginine after post-translational methylation [19] and is a competitive inhibitor of NO synthase, while SDMA, a structural isomer of ADMA, is neither an NO synthase substrate nor inhibitor, but mainly inhibits the cellular reception of the NO precursor arginine [20]. Hence, the common effect of these two agents, especially in the presence of pathologically elevated concentrations of ADMA and SDMA, is a reduction of NO synthesis [20]. Accumulating evidence shows that both ADMA and SDMA have predictive value regarding mortality and CVD events in observational studies [18,21]. Their effects may contribute to endothelial dysfunction and atherogenesis [22,23]. Concordantly, elevated ADMA and SDMA levels were observed in hypertensive children and adolescents [24]. SDMA is mainly excreted through the renal system, alongside approximately 20% of ADMA [25]. Therefore, in case of renal failure, both ADMA and SDMA levels rise in blood serum, with SDMA rising to a greater extent [26]. Significant correlations of SDMA [24] and ADMA [22] with renal function were confirmed, while elevated levels may occur even before a reduction of the glomerular filtration rate (GFR) can be detected [22]. Taken together, the modulation of NO metabolism by L-homoarginine, ADMA and SDMA may impact endothelial dysfunction and blood pressure by affecting endothelial cell and vascular smooth muscle cell function, a hypothesis that is evaluated in the present investigation (see Figure 1).

Altogether, there is a knowledge gap regarding the link between homoarginine, ADMA or SDMA and arterial hypertension as well as endothelial dysfunction. Hence, we report measurements of these parameters derived from the Styrian Vitamin D Hypertension Trial [27], a cohort of hypertensive patients, to address the question whether serum concentrations of these NO synthesis markers are associated with blood pressure values and measures of endothelial dysfunction. We hypothesize that there is a significant correlation between blood pressure values and NO synthesis markers.

## 2. Methods

This study was a post-hoc analysis based on the data of participants who underwent screening procedures for the double blind, placebo-controlled Styrian Vitamin D Hypertension Trial [registered at www.clinicaltrialsregister.eu (EudraCT Number 2009-018125-70) and clinicaltrials.gov (Identifier NCT02136771)] [27]. In this study, we investigated clinical and laboratory biomarkers in patients with arterial hypertension. The study was performed at the Medical University of Graz, Austria, and was approved by the local ethics committee. All study participants (*n* = 514) provided written informed consent, were older than 18 years and had a 25-hydroxy-vitamin D serum concentration below 30 ng/mL (=75 nmol/L), as well as arterial hypertension. The latter was assessed with 24 h ambulatory blood pressure monitoring and was defined as a blood pressure of systolic >140 mmHg or diastolic >90 mmHg, mean 24-h blood pressure of systolic >125 mm Hg or diastolic >80 mmHg, home blood pressure of systolic >130 mm Hg or diastolic >85 mmHg or ongoing antihypertensive treatment. Further details of the study design and methods were published elsewhere [27,29,30].

SDMA, ADMA and homoarginine were measured from frozen serum (−70 °C) by high-performance liquid chromatography (HPLC, Agilent, Palo Alto, CA, USA) with solid phase extraction and precolumn derivatization [31]. Within-day coefficients of variation (CVs) for SDMA were 4.6% (0.60 µmol/L) and 1.9% (1.0 µmol/L), and between-day CVs were 9.8% (0.60 µmol/L) and 6.1% (1.0 µmol/L), respectively. Within-day CVs for ADMA were 3.1% (0.62 µmol/L) and 1.0% (2.0 µmol/L), and between-day CVs were 9% (0.62 µmol/L) and 2.2% (2.0 µmol/L), respectively. Within-day CVs for homoarginine were 4.7% (1.21 µmol/L) and 2.2% (3.53 µmol/L), and between-day CVs were 7.9% (1.25 µmol/L) and 6.8% (3.66 µmol/L), respectively.

Data were reported as mean and standard deviation for continuous variables with normal distribution and as median with interquartile range for continuous variables with non-normal distribution. Categorical data were presented as percentages. The cohort was stratified into quartiles of homoarginine, ADMA and SDMA levels. To assess differences of variables between quartiles we performed an analysis of variance (ANOVA). To assess whether there was a trend across the quartiles, a trend test was performed using linear degree. Differences in categorical variables were assessed using the chi-squared test with p for linear by linear test. To investigate the relationships of homoarginine, ADMA and SDMA levels with PWV and blood pressure values, Pearson’s correlation coefficient was used and linear regression analyses with adjustments for age, gender and GFR. Non-normally distributed variables were logarithmically transformed before use in parametric procedures. *p*-values of <0.05 were considered statistically significant. The statistical software package used was IBM^®^ SPSS^®^ Statistics Version 26 (IBM Corporation, Armonk, NY, USA).

## 3. Results

Among a total of 514 study participants, 509 had available values of homoarginine, ADMA, SDMA and 24 h blood pressure measurements and were thus included in the current analysis. The mean age was 61.2 ± 10.5 years, mean PWV was 8.6 ± 2.4 m/s, mean systolic blood pressure was 127.5 ± 13.8 mmHg and mean 24 h diastolic blood pressure was 76.4 ± 9.5 mmHg. Patient characteristics were stratified by quartiles of homoarginine, ADMA and SDMA, as shown in Table 1, Table 2 and Table 3.

**Figure 2:**
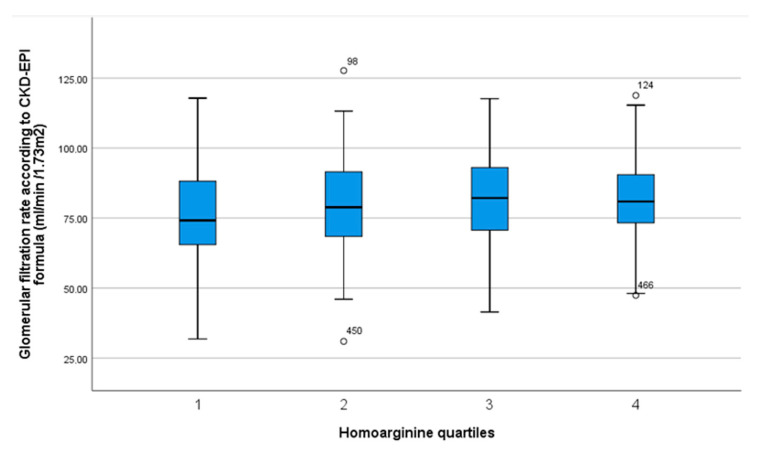
Box plot with median, interquartile range, total range and outliers of glomerular filtration rate according to CKD-EPI formula stratified by quartiles of homoarginine serum concentration.

**Figure 3:**
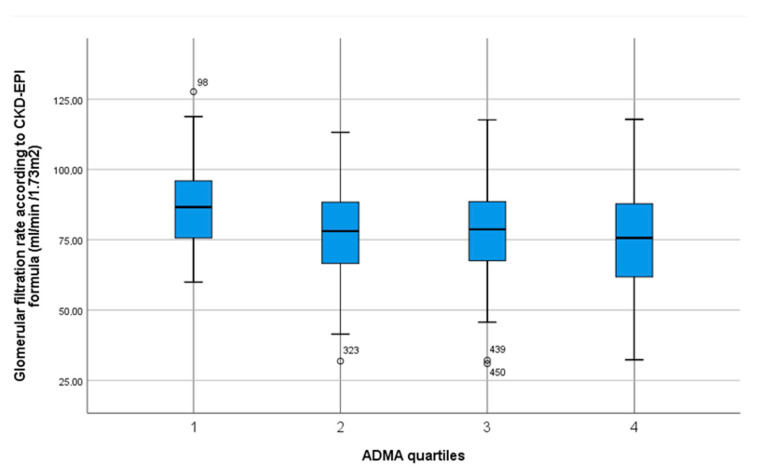
Box plot with median, interquartile range, total range and outliers of glomerular filtration rate according to CKD-EPI formula stratified by quartiles of ADMA serum concentration.

**Figure 4:**
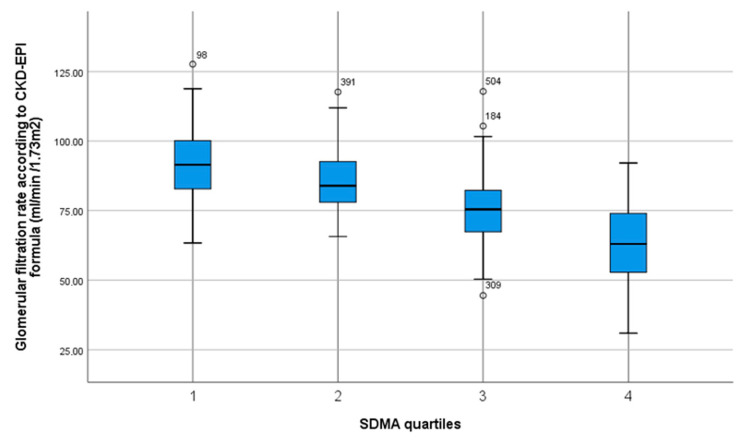
Box plot with median, interquartile range, total range and outliers of glomerular filtration rate according to CKD-EPI formula stratified by quartiles of SDMA serum concentration.

Bivariate analysis revealed lower homoarginine levels in females. Homoarginine, ADMA and SDMA differed by BMI as well as age. No significant differences were found between quartiles of homoarginine and ADMA regarding medication, except ACE inhibitors regarding homoarginine levels and beta-blockers regarding ADMA levels. However, for SDMA quartiles, there were multiple significant differences regarding antihypertensive drug intakes (see Table 3). Higher quartiles of ADMA and SDMA were inversely correlated with kidney function, whereas homoarginine quartiles were positively correlated with this parameter (Figure 2, Figure 3 and Figure 4). Regarding lipid metabolism, HDL was significant lower across higher homoarginine and ADMA quartiles, but no other significant trends were observed. Nocturnal blood pressure dipping and heart rate were significantly lower in higher SDMA quartiles.

**Figure 5:**
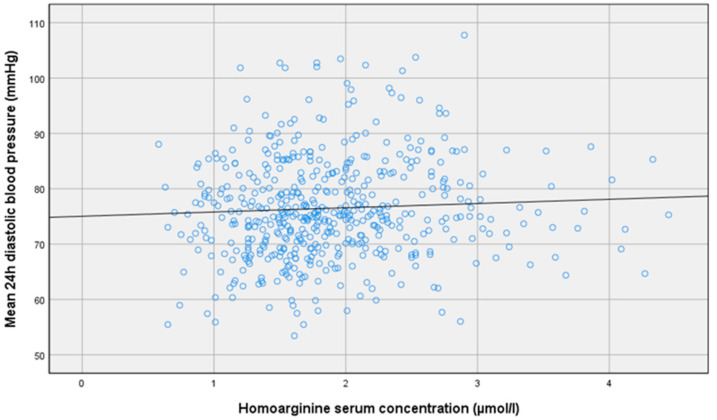
Pearson correlation analyses of homoarginine with mean 24 h diastolic blood pressure: r = 0.1; *p =* 0.02.

Analyzing continuous variables for our primary outcome measures (Table 4), bivariate correlations reached statistical significance only between homoarginine and mean 24 h diastolic blood pressure with a Pearson correlation coefficient of 0.1 (Figure 5). However, after adjustment for age, gender and GFR according to the CKD-EPI formula, regression analysis was no longer significant between mean 24 h diastolic blood pressure and homoarginine (*p =* 0.43).

## 4. Discussion

In this retrospective analysis of the Styrian Hypertension Study, we were not able to show a significant and independent association of ADMA, SDMA and homoarginine with blood pressure values and PWV.

Our findings of no significant association of NO synthesis markers with PWV and 24 h blood pressure values significantly add to the sparse and controversial literature on this topic. In the light of all evidence, results regarding the link between NO synthesis markers and arterial hypertension remain heterogeneous, as some studies showed a positive [32], but others [33] a negative, association between homoarginine and blood pressure. Differences in study populations and study designs may explain these partially inconsistent results. Given that the NO synthesis markers we evaluated in our study were previously associated with cardiovascular outcomes, our findings may suggest that endothelial dysfunction and blood pressure are not the main drivers for these associations. Alternatively, it must also be considered that previously observed associations between NO synthesis markers and clinical endpoints may not reflect causal relationships and may be due to unconsidered or unmeasured confounding. Nevertheless, as potentially treatable, modifiable cardiovascular risk factors, homoarginine, ADMA and SDMA are increasingly attracting attention as potential drug targets. Knockout of arginine:glycine amidinotransferase and therefore inhibition of homoarginine synthesis led to increased cardiovascular events in animal models, with supplementation of homoarginine shown to prevent these events [34,35]. Thus, supplementation of homoarginine, which also reduces ADMA synthesis by inhibiting the enzyme arginine methyltransferase and additionally improves NO bioavailability by being not only a substrate but also a competitor with ADMA for NO synthase [36], may be a new approach in CVD management. Concordantly, intravenous low-dose ADMA resulted in a reduction of heart rate and cardiac output and an elevation of vascular resistance and mean blood pressure [37]. However, these results remained inconsistent, as an inverse correlation between ADMA and blood pressure values was described in another study [32]. Data on therapeutic strategies to modulate NO synthesis markers are lacking and a specific ADMA- or SDMA-modifying agent is not available. Associations of several drugs, including angiotensin-converting enzyme inhibitors, angiotensin receptor blockers and metformin, with reduced ADMA-levels were reported, although their underlying effects are unknown and such data need confirmation by additional studies [36].

Apart from our null findings on the primary outcome measures in our investigation, we confirmed previously established associations of NO synthesis markers with some clinical and laboratory characteristics. For example, levels of ADMA and SDMA were reported to increase with age, while homoarginine decreases [38], which is in line with our results. Pathophysiological mechanisms and consequences in chronic kidney disease (CKD) regarding NO metabolism are being increasingly investigated. We observed a significant positive correlation between homoarginine and GFR, and inverse correlations of ADMA and SDMA with GFR. Our findings are in line with previous investigations showing an increase in serum concentrations in case of renal failure, especially for SDMA [26], thus supporting the reliability of our laboratory measurements. The highly significant association of SDMA with renal function was further confirmed by a meta-analysis [21]. Elevated ADMA levels were shown to occur even before a reduction in GFR [22].

Our study should be interpreted within the context of its strengths and limitations. Antihypertensive treatment rates between quartiles appeared heterogeneous and may have a significant impact on blood pressure values. However, bivariate correlations did not indicate a significant association between blood pressure values and homoarginine, ADMA or SDMA. Due to multiple testing in this analysis, statistical significances may result by chance but our null findings obviate the need to consider p-value adjustments for multiple testing. Further, NO synthesis parameters do not only derive from metabolism, but can also be derived from or modified by food intake, and no details regarding diet were assessed. However, we assumed that the impact of homoarginine as well as ADMA and SDMA derived from food intake on serum levels was small and that the majority derives from endogenous synthesis. A further limitation is that we did not perform more in-depth measurements of vascular function, such as brachial artery dilation tests. Based on the data of the Styrian Vitamin D Hypertension Trial, the patient cohort was a selected population and results may not easily transfer to other population groups. A strength of this study is the simultaneous performance of blood sampling and 24 h blood pressure measurement.

In conclusion, this analysis did not reveal a significant association between NO synthesis markers and blood pressure values or PWV in hypertensive adults. These results suggest that the associations of these parameters with adverse outcomes may not be mediated by hypertension and/or endothelial dysfunction. Based on the current knowledge, homoarginine supplementation appears to be safe and well tolerated and, thus, may represent a new therapeutic strategy in CVD management. Given that NO synthesis markers may represent a modifiable cardiovascular risk factor in CVD, the current data may further encourage larger RCTs to shed light on the potential causal role of homoarginine, ADMA and SDMA in CVD, and to achieve a better understanding of the processes leading to variations in levels of those parameters in specific patient populations.

## Figures and Tables

**Table 1:** Clinical and laboratory characteristics according to quartiles of homoarginine.

**Homoarginine Quartiles [µmol/L]**	**0.58–1.45** ***n* = 127**	**1.46–1.77** ***n* = 131**	**1.78–2.23** ***n* = 124**	**2.24–4.45** ***n* = 127**	***p* Value Trend**
Female [%]	71.7	56.5	44.4	36.2	*p* < 0.001 *
Age [years]	63.5 ± 9.9	61.1 ± 1.1	60.9 ± 10.3	59.2 ± 10.5	*p* = 0.002 *
BMI [kg/m^2^]	28.6 ± 5.4	29.4 ± 4.8	30.0 ± 5.1	30.4 ± 5.1	*p* = 0.004 *
**Laboratory results**					
Glomerular filtration rate (CKD-EPI formula)	75.0 ± 18.6	80.3 ± 17.5	80.8 ± 17.3	81.7 ± 14.9	*p* = 0.022 *
HbA1c [mmol/mol]	44.9 ± 12.6	43.6 ± 11.9	41.9 ± 9.8	41.8 ± 12.1	*p* = 0.29
Total cholesterol [mg/dL]	206 (169.8–228)	198 (163.8–231)	199 (171–224.5)	186 (162–217)	*p* = 0.09
HDL [mg/dL]	60.5 (51.8–75)	55 (46–69)	55.5 (45–68)	54 (44–65)	*p* = 0.006 *
LDL [mg/dL]	114.5 (88.5–143.3)	112.5 (82.3–148.3)	115 (90–140)	104 (87–133.5)	*p* = 0.81
Triglycerides [mg/dL]	99 (70.8–142.5)	110 (76.5–145.3)	112 (78.3–164.5)	117 (80–169)	*p* = 0.06
**Medication [%]**					
Beta blockers	46.8	49.6	50	50	*p* = 0.95
ACE inhibitors	38.1	35.9	24.2	40.5	*p* = 0.035 *
Angiotensin II receptor antagonists	30.2	34.4	34.7	30.2	*p* = 0.78
Calcium channel antagonists	27.0	24.4	29.8	23.0	*p* = 0.10
Diuretics	48.4	45.0	41.9	41.3	*p* = 0.65
Aldosterone antagonists	4.8	3.1	4.0	4.0	*p* = 0.92
Other antihypertensive drugs	12.0	16.0	11.3	17.5	*p* = 0.42
Glucocorticoids	3.2	1.5	4.9	1.6	*p* = 0.32
Pulse wave velocity [m/sec]	8.9 ± 2.5	8.8 ± 2.2	8.0 ± 2.1	8.5 ± 2.5	*p* = 0.03 *
Mean 24 h systolic blood pressure [mm Hg]	125.2 ± 14.9	127.9 ± 13.5	127.9 ± 13.4	129.2 ± 13.4	*p* = 0.03 *
Mean 24 h diastolic blood pressure [mm Hg]	74.9 ± 8.7	75.3 ± 8.9	77.1 ± 9.4	78.2 ± 10.5	*p* = 0.002 *
Mean 24 h heart rate [bpm]	71.6 ± 9.5	72.5 ± 10.5	70.6 ± 9.0	72.8 ± 10.0	*p* = 0.68
Mean night systolic blood pressure [mm Hg]	116.4 ± 16.9	116.8 ± 16.3	115.8 ± 19.6	117.6 ± 14.7	*p* = 0.72
Mean night diastolic blood pressure [mm Hg]	66.9 ± 9.0	66.6 ± 9.5	68.5 ± 9.4	68.6 ± 9.3	*p* = 0.07
Mean night heart rate [bpm]	64.4 ± 9.3	65.7 ± 9.7	63.8 ± 9.6	64.8 ± 8.9	*p* = 0.86
Mean Systolic Dipping [%]	−11.0 ± 9.5	−11.9 ± 9.6	−11.7 ± 8.0	−12.2 ± 9.2	*p* = 0.39
Mean Diastolic Dipping [%]	−15.6 ± 9.1	−15.3 ± 10.0	−15.1 ± 8.6	−15.7 ± 8.8	*p* = 0.98

Data are shown as ranges, percentage and medians (25th to 75th percentile) and as means ± standard deviation. Analysis of variance (ANOVA) with p for trend and Chi Square test with p for linear by linear test was calculated. Significant results are marked with an asterisk (*). ACE = angiotensin converting enzyme; BMI = body mass index; CKD-EPI = Chronic Kidney Disease Epidemiology Collaboration; HbA1c = glycated hemoglobin; HDL = High Density Lipoprotein; LDL = Low Density Lipoprotein.

**Table 2:** Clinical and laboratory characteristics according to quartiles of ADMA.

**ADMA Quartiles [µmol/L]**	**0.47–0.64** ***n* = 127**	**0.65–0.70** ***n* = 138**	**0.71–0.77** ***n* = 125**	**0.78–0.98** ***n* = 119**	***p* Value Trend**
*Female [%]*	49.6	47.4	56.8	56.3	*p* = 0.33
*Age [years]*	57.6 ± 11.4	60.9 ± 9.8	63.3 ± 9.9	63.2 ± 10.1	*p* < 0.001 *
*BMI [kg/m^2^]*	28.8 ± 4.7	29.5 ± 4.7	29.3 ± 4.8	30.8 ± 6.0	*p* = 0.004 *
***Laboratory results***					
*Glomerular filtration rate (CKD-EPI formula)*	87.2 ± 14.5	76.9 ± 17.1	77.8 ± 16.5	75.0 ± 18.7	*p* < 0.001 *
*HbA1c [mmol/mol]*	42.7 ± 12.0	42.7 ± 11.7	44.6 ± 11.5	44.5 ± 11.4	*p* = 0.12
*Total cholesterol [mg/dL]*	200 (171–231)	205 (172–229.5)	184 (158–219.5)	194.5 (164–223)	*p* = 0.44
*HDL [mg/dL]*	58 (47–75)	56 (45–66.5)	57 (48–68)	54 (42.8–67)	*p* = 0.03 *
*LDL [mg/dL]*	115 (95–140.5)	120 (93–143)	99 (80–137)	109.5 (88–141.3)	*p* = 0.31
*Triglycerides [mg/dL]*	108 (73–138)	115 (77–175)	94 (70.5–139.5)	122.5 (84.8–166.5)	*p* = 0.24
***Medication [%]***					
*Beta blockers*	41.3	43.8	55.2	57.1	*p* = 0.02 *
*ACE inhibitors*	33.3	38.0	33.6	33.6	*p* = 0.83
*Angiotensin II receptor antagonists*	35.7	27.7	34.4	31.9	*p* = 0.53
*Calcium channel antagonists*	22.2	21.2	32.8	28.6	*p* = 0.11
*Diuretics*	38.1	42.3	44.0	52.9	*p* = 0.12
*Aldosterone antagonists*	4.0	4.4	3.2	4.2	*p* = 0.97
*Other antihypertensive drugs*	11.1	16.8	14.4	14.4	*p* = 0.63
*Glucocorticoids*	1.6	1.5	3.2	5.0	*p* = 0.28
*Pulse wave velocity [m/sec]*	8.2 ± 2.0	8.7 ± 2.1	8.9 ± 2.4	8.4 ± 3.0	*p* = 0.31
*Mean 24 h systolic blood pressure [mm Hg]*	127.7 ± 13.2	126.7 ± 13.7	126.4 ± 13.8	129.5 ± 14.7	*p* = 0.38
*Mean 24 h diastolic blood pressure [mm Hg]*	78.7 ± 10.0	76.1 ± 9.7	75.1 ± 8.8	75.6 ± 9.1	*p* = 0.007 *
*Mean 24 h heart rate [bpm]*	72.2 ± 9.3	72.0 ± 10.4	71.8 ± 9.5	71.6 ± 10.0	*p* = 0.61
*Mean night systolic blood pressure [mm Hg]*	116.7 ± 19.0	115.1 ± 14.3	116.5 ± 19.5	118.8 ± 14.0	*p* = 0.28
*Mean night diastolic blood pressure [mm Hg]*	69.1 ± 9.2	67.2 ± 9.7	67.1 ± 9.6	67.2 ± 8.4	*p* = 0.12
*Mean night heart rate [bpm]*	64.8 ± 8.8	64.6 ± 10.0	64.6 ± 9.0	64.8 ± 9.6	*p* = 0.99
*Mean Systolic Dipping [%]*	−11.8 ± 9.6	−13.1 ± 8.4	−10.9 ± 9.2	−10.8 ± 9.2	*p* = 0.17
*Mean Diastolic Dipping [%]*	−16.1 ± 9.2	−15.9 ± 9.0	−14.8 ± 9.3	−14.8 ± 9.2	*p* = 0.17

Data are shown as ranges, percentage and medians (25th to 75th percentile) and as means ± standard deviation. Analysis of variance (ANOVA) with p for trend and Chi Square test with p for linear by linear test was calculated. Significant results are marked with an asterisk (*). ACE = angiotensin converting enzyme; BMI = body mass index; CKD-EPI = Chronic Kidney Disease Epidemiology Collaboration; HbA1c = glycated hemoglobin; HDL = High Density Lipoprotein; LDL = Low Density Lipoprotein.

**Table 3:** Clinical and laboratory characteristics according to quartiles of SDMA.

**SDMA Quartiles [µmol/L]**	**0.44–0.60** ***n* = 127**	**0.61–0.69** ***n* = 138**	**0.70–0.79** ***n* = 124**	**0.80–1.73** ***n* = 120**	***p* Value Trend**
*Female [%]*	52.0	50.0	42.3	50.0	*p* = 0.57
*Age [years]*	56.6 ± 10.2	59.4 ± 10.8	61.6 ± 10.2	67.6 ± 7.4	*p* < 0.001 *
*BMI [kg/m^2^]*	30.6 ± 5.3	29.6 ± 5.4	28.7 ± 4.2	29.6 ± 5.3	*p* = 0.07
***Laboratory results***					
*Glomerular filtration rate (CKD-EPI formula)*	92.0 ± 13.4	85.7 ± 11.1	75.9 ± 13.3	62.5 ± 14.9	*p* < 0.001 *
*HbA1c [mmol/mol]*	46.4 ± 15.3	42.3 ± 9.9	41.7 ± 9.7	43.9 ± 10.6	*p* = 0.08
*Total cholesterol [mg/dL]*	187 (165–218)	206.5 (171.8–234.5)	205 (169–229)	186 (157–220)	*p* = 0.43
*HDL [mg/dL]*	54 (44.8–66)	57 (48–73)	57 (48–69)	56 (44–69)	*p* = 0.32
*LDL [mg/dL]*	108 (84.5–137)	122 (92.5–148.5)	121 (93–145)	101 (80.5–131.5)	*p* = 0.17
*Triglycerides [mg/dL]*	118 (81–176)	109 (73–138)	106 (78–144)	104 (73–169)	*p* = 0.16
***Medication [%]***					
*Beta blockers*	43.3	43.9	48.4	55.8	*p* = 0.27
*ACE inhibitors*	33.9	41.3	23.8	39.2	*p* = 0.02 *
*Angiotensin II receptor antagonists*	31.5	26.1	41.8	30.8	*p* = 0.054
*Calcium channel antagonists*	26.0	19.6	33.6	25.8	*p* = 0.09
*Diuretics*	39.4	39.9	40.2	58.3	*p* = 0.01 *
*Aldosterone antagonists*	2.4	0.0	3.3	10.8	*p* < 0.001 *
*Other antihypertensive drugs*	10.2	13.0	12.3	21.8	*p* = 0.049 *
*Glucocorticoids*	0.0	5.1	0.8	5.0	*p* = 0.02 *
*Pulse wave velocity [m/s]*	8.3 ± 2.0	8.4 ± 1.9	8.7 ± 2.7	8.9 ± 2.9	*p* = 0.03 *
*Mean 24 h systolic blood pressure [mm Hg]*	128.6 ± 13.2	127.5 ± 13.3	126.2 ± 12.8	127.8 ± 16.2	*p* = 0.49
*Mean 24 h diastolic blood pressure [mm Hg]*	78.6 ± 9.5	77.0 ± 8.9	76.4 ± 9.8	73.2 ± 9.1	*p* < 0.001 *
*Mean 24 h heart rate [bpm]*	74.7 ± 9.8	71.2 ± 9.5	71.6 ± 10.4	70.1 ± 8.9	*p* = 0.001 *
*Mean night systolic blood pressure [mm Hg]*	116.2 ± 17.4	116.7 ± 15.7	115.8 ± 13.7	118.2 ± 20.4	*p* = 0.45
*Mean night diastolic blood pressure [mm Hg]*	69.1 ± 9.4	68.0 ± 9.6	67.1 ± 8.1	66.2 ± 9.9	*p* = 0.01 *
*Mean night heart rate [bpm]*	67.2 ± 9.5	64.2 ± 8.8	63.4 ± 10.0	63.8 ± 8.9	*p* = 0.003 *
*Mean Systolic Dipping [%]*	−12.8 ± 8.6	−12.6 ± 8.7	−11.5 ± 9.1	−9.6 ± 9.9	*p* = 0.003 *
*Mean Diastolic Dipping [%]*	−16.5 ± 8.9	−16.2 ± 9.0	−15.2 ± 8.9	−13.6 ± 9.7	*p* = 0.008 *

Data are shown as ranges, percentage and medians (25th to 75th percentile) and as means ± standard deviation. Analysis of variance (ANOVA) with p for trend and Chi Square test with p for linear by linear test was calculated. Significant results are marked with an asterisk (*). ACE = angiotensin converting enzyme; BMI = body mass index; CKD-EPI = Chronic Kidney Disease Epidemiology Collaboration; HbA1c = glycated hemoglobin; HDL = High Density Lipoprotein; LDL = Low Density Lipoprotein.

**Table 4:** Pearson correlation analyses of NO synthesis markers with PWV and 24 h blood pressure and heart rate values.

*Bivariate Correlations*	Homoarginine	ADMA	SDMA
**Pulse wave velocity [m/s]**	r = −0.09 *p* = 0.057	r = −0.1 *p* = 0.83	r = −0.01 *p* = 0.86
**Mean 24 h systolic blood pressure [mm Hg]**	r = 0.07 *p* = 0.11	r = 0.02 *p* = 0.59	r = 0.02 *p* = 0.61
**Mean 24 h diastolic blood pressure [mm Hg]**	r = 0.1 *p* = 0.02 *	r = −0.01 *p* = 0.86	r = −0.02 *p* = 0.70
**Mean 24 h heart rate [bpm]**	r = 0.01 *p* = 0.89	r = 0.002 *p* = 0.96	r = −0.01 *p* = 0.89
**Mean daytime systolic blood pressure [mm Hg]**	r = 0.06 *p* = 0.18	r = 0.03 *p* = 0.54	r = −0.03 *p* = 0.56
**Mean daytime diastolic blood pressure [mm Hg]**	r = 0.07 *p* = 0.12	r = −0.01 *p* = 0.91	r = −0.02 *p* = 0.73
**Mean night systolic blood pressure [mm Hg]**	r = 0.02 *p* = 0.68	r = 0.01 *p* = 0.85	r = 0.01 *p* = 0.82
**Mean night diastolic blood pressure [mm Hg]**	r = 0.06 *p* = 0.19	r = −0.01 *p* = 0.76	r = −0.02 *p* = 0.67
**Mean Systolic Dipping [%]**	r = −0.05 *p* = 0.26	r = −0.01 *p* = 0.75	r = −0.01 *p* = 0.87
**Mean Diastolic Dipping [%]**	r = −0.03 *p* = 0.51	r = −0.01 *p* = 0.78	r = −0.01 *p* = 0.90

Significant results are marked with an asterisk (*).

## Abbreviations

ACE	Angiotensin converting enzyme
ADMA	Asymmetric dimethylarginine
AGXT2	Alanine-glyoxylate aminotransferase 2
ANOVA	Analysis of variance
BMI	Body mass index
CD	Cardiovascular disease
cGMP	Cyclic guanosinmonophosphate
CKD-EPI	Chronic Kidney Disease Epidemiology Collaboration
CV	Coefficient of variation
DDAH I/II	Dimethylarginine dimethylaminohydrolase I/II
GFR	Glomerular filtration rate
HbA1c	Glycated hemoglobin
HDL	High Density Lipoprotein
HPLC	High-performance liquid chromatography
LDL	Low Density Lipoprotein
NO	Nitric oxide
PRMT I/II	Protein arginine methyltransferases I/II
PWV	Pulse wave velocity
SDMA	Symmetric dimethylarginine
